# The zinc proteome of SARS-CoV-2

**DOI:** 10.1093/mtomcs/mfac047

**Published:** 2022-06-29

**Authors:** Claudia Andreini, Fabio Arnesano, Antonio Rosato

**Affiliations:** Consorzio Interuniversitario di Risonanze Magnetiche di Metallo Proteine, Via Luigi Sacconi 6, 50019 Sesto Fiorentino, Italy; Department of Chemistry and Magnetic Resonance Center (CERM), University of Florence, Via Luigi Sacconi 6, 50019 Sesto Fiorentino, Italy; Department of Chemistry, University of Bari “Aldo Moro,” Via Orabona 4, 70125 Bari, Italy; Consorzio Interuniversitario di Risonanze Magnetiche di Metallo Proteine, Via Luigi Sacconi 6, 50019 Sesto Fiorentino, Italy; Department of Chemistry and Magnetic Resonance Center (CERM), University of Florence, Via Luigi Sacconi 6, 50019 Sesto Fiorentino, Italy

**Keywords:** Zinc, SARS-CoV-2, Zinc finger motifs, Bioinformatics, Metalloproteins

## Abstract

Zinc is an essential element for human health. Among its many functions, zinc(II) modulates the immune response to infections and, at high concentrations or in the presence of ionophores, inhibits the replication of various RNA viruses. Structural biology studies on severe acute respiratory syndrome coronavirus 2 (SARS-CoV-2) revealed that zinc(II) is the most common metal ion that binds to viral proteins. However, the number of zinc(II)-binding sites identified by experimental methods is far from exhaustive, as metal ions may be lost during protein purification protocols. To better define the zinc(II)-binding proteome of coronavirus, we leveraged the wealth of deposited structural data and state-of-the-art bioinformatics methods. Through this *in silico* approach, 15 experimental zinc(II) sites were identified and a further 22 were predicted in Spike, open reading frame (ORF)3a/d, ORF8, and several nonstructural proteins, highlighting an essential role of zinc(II) in viral replication. Furthermore, the structural relationships between viral and eukaryotic sites (typically zinc fingers) indicate that SARS-CoV-2 can compete with human proteins for zinc(II) binding. Given the double-edged effect of zinc(II) ions, both essential and toxic to coronavirus, only the complete elucidation of the structural and regulatory zinc(II)-binding sites can guide selective antiviral strategies based on zinc supplementation.

## Graphical Abstract

**Graphical Abstract:**
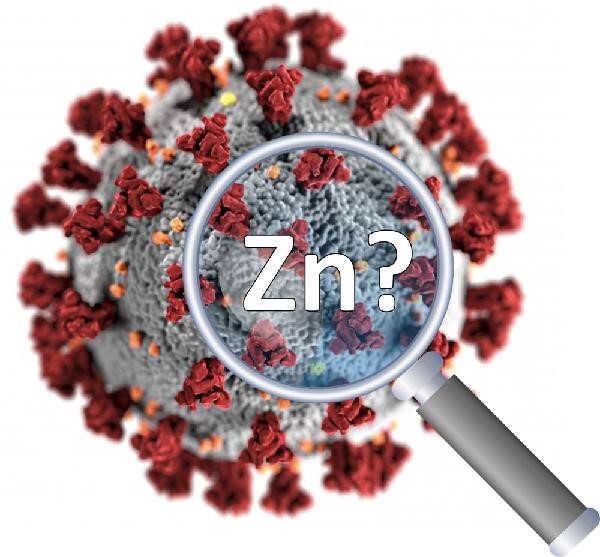
A large share of SARS-Cov-2 proteins contain potential zinc(II)-binding sites, some with structural support and others predicted by proteome-wide bioinformatic analysis, which show a similarity to eukaryotic zinc finger sites. Open questions and implications for the treatment of COVID-19 are presented.

## Introduction

One of the most intriguing and least debated aspects of infection is the maturation of the viral proteins through post-translational modifications. In this regard, an essential maturation process is the loading of metal ions, which must be supplied by the host. For this reason, the host has developed a protective mechanism, known as nutritional immunity,^1^ which inhibits the growth of pathogens by limiting the uptake of specific metal ions that are crucial nutrients.

Iron and zinc perform many physiological functions in almost all living organisms and are also necessary for viral growth. Circulating concentrations of these minerals decline rapidly and dramatically with the inflammation associated with infection. The decline in iron and zinc is thought to starve invading pathogens of these essential elements, limiting disease progression and severity.^2^ It is therefore not surprising that massive amounts of calprotectin (S100A8/S100A9), a zinc(II)-sequestering protein, have been found in the plasma of patients developing severe COVID-19.^3^ Calprotectin is upregulated in zinc-limiting conditions and functions by binding two zinc(II) ions, thereby chelating and starving microbes of this essential mineral.^4^ Thus, preventing the maturation of viral zinc(II)-binding proteins seems a viable therapeutic strategy against COVID-19. In this context, the use of selective zinc(II) ionophores has been explored.^5^ Another proposed strategy is to target the zinc fingers in viral metalloenzymes^6^; a few compounds that replace zinc(II) by another metal ion have entered clinical trials.^7^ Bismuth(III) ions strikingly compete with the zinc(II) ions of the viral helicase (nonstructural protein; Nsp13), leading to compromised enzyme activity and severe deficiencies in viral replication of severe acute respiratory syndrome coronavirus (SARS-CoV)^8^ and SARS-CoV-2.^9^

On the other side of the coin, when their concentration reaches toxic levels, metal ions can inhibit protein function. *In vitro* studies have demonstrated a number of mechanisms by which zinc interferes with a viral replication cycle, such as inhibition of viral uncoating, viral genome transcription, viral protein translation, and polyprotein processing.^10^^,^^11^ In the case of coronaviruses, it is known that zinc(II) can inhibit essential proteins, such as RNA-dependent RNA-polymerase (RdRp, Nsp12),^12^ chymotrypsin-like main protease (3CLpro or Mpro, Nsp5),^13^^,^^14^ and papain-like protease (PLpro, Nsp3).^15^^,^^16^ These three enzymes play a key role in viral replication and are conserved in SARS-CoV and SARS-CoV-2.^17^ However, it is unclear whether it is possible to reach *in vivo* the zinc(II) concentration levels used in *in vitro* experiments without negatively affecting the host.^18^

Based on the earlier-mentioned evidences and the established zinc(II)-mediated immunomodulatory response, it has been hypothesized that zinc supplementation may be of benefit for prophylaxis and treatment of COVID-19.^19–22^ The potential impact of zinc supplementation on COVID-19 pathogenesis has been evaluated as well.^23^^,^^24^ However, the COVID-19 Treatment Guidelines of NIH discourage zinc supplementation above the recommended dietary allowance (11 mg daily for men and 8 mg for nonpregnant women; https://www.covid19treatmentguidelines.nih.gov/therapies/supplements/zinc/), except in clinical trials (with a maximum dose of 50 mg of elemental zinc twice daily; https://clinicaltrials.gov/ct2/results?cond=COVID-19&term=zinc+supplement).

The earlier-mentioned considerations on the double-edged effect of zinc ions point out that molecular mechanisms of zinc(II) homeostasis at the host–pathogen interface must be deeply understood to devise proper and selective antiviral strategies. In this work, we leveraged the wealth of deposited structural data^25^ and state-of-the-art bioinformatics methods^26–28^ to predict which SARS-CoV-2 proteins need zinc ions to perform their function. In other words, we defined, by an *in silico* approach, the zinc(II) proteome of SARS-CoV-2. Furthermore, we analysed the structural relationships between viral and eukaryotic zinc(II)-binding sites.

## Materials and methods

As first step, we created two libraries by applying the approach developed in Valasatava *et al.*^26^ The first library contained hidden Markov model (HMM) profiles^29^ of zinc(II)-binding domains (Pfam library) whereas the second contained HMM profiles of zinc(II)-binding structural motifs (Motif library). As described in Andreini *et al.*^28^ the Pfam domain library was built by merging two lists: (i) a list of Pfam domains annotated as zinc(II) binding, retrieved by mining the text of the annotations in the Pfam database^29^ and (ii) a list of zinc(II)-binding patterns derived from the analysis of the sequence of zinc(II)-binding proteins with known 3D structure available in MetalPDB,^30^ where each HMM profile is associated with the positions of the protein residues responsible for zinc(II) binding. The procedure resulted in a set of 573 Pfam profiles: 541 with an associated zinc(II)-binding pattern, and an additional 32 simply annotated as zinc(II)-binding domains.

The library of zinc(II)-binding structural motifs was created by splitting into fragments the zinc(II)-binding sites stored in MetalPDB as of May 2020. Only one representative was kept for sites that, although found in different Protein Data Bank (PDB) structures, fall in the same position within the same protein domain.^30^^,^^31^ Only motifs containing at least one zinc(II)-binding residue were kept. In addition, we manually removed zinc(II)-binding sites that are not physiologically relevant [e.g. spurious sites or zinc(II)-substituted structures] by inspecting the literature, as follows: if the relevant article(s) did not describe the function of the zinc(II) ion and the experimental section reported that it was present in the purification or crystallization buffer, then we annotated the site as spurious. We further discarded all sites with no donor atoms from the protein. This procedure resulted in a library of 3298 zinc(II)-binding motifs derived from 2499 zinc(II)-binding sites.

The zinc(II) proteome of SARS-CoV-2 was retrieved by applying the hmmscan tool (http://hmmer.org/) to search each protein sequence for the profiles contained in the two libraries. The occurrence of a potential zinc(II)-binding site was identified if at least one of the following conditions was verified: (i) the profiles of all the fragments of a given site matched the viral sequence with an *e*-value lower than 10^−3^ and the corresponding zinc(II)-binding residues (i.e. protein residues that provide donor atoms) are conserved in the sequence; (ii) the profile of a domain with an associated zinc(II)-binding pattern matched the sequence with an *e*-value lower than 10^−5^ and the pattern is conserved in the sequence; and (iii) the profile of a domain with no associated pattern matched the sequence with an *e*-value lower than 10^−7^. This procedure (and in particular the selected *e*-value thresholds) was optimized in Valasatava *et al.*^26^ These predictions were integrated by adding the experimentally determined zinc sites in the PDB^32^ and with sites that have annotated as zinc(II) binding in the UniProt database.^33^ A similar analysis was performed on the proteomes of SARS-CoV and Middle East respiratory syndrome coronavirus (MERS-CoV).

For structure modeling, we downloaded the 3D structural models generated by AlphaFold^34^ from the corresponding database at EBI.^35^ For each viral protein, the metal-binding residues predicted by our approach were identified in the downloaded model and a zinc(II) ion was put at the geometric center of the donor atoms.

We input each zinc site with an available 3D structure or structural model to the MetalS^3^ program^36^ to identify zinc(II) sites with a similar local structure in the entire MetalPDB database, using default parameters.

## Results and discussion

### Identification of zinc sites in SARS-CoV-2 proteins

The initial two-thirds of the SARS-CoV-2 genome encode the two overlapping polyproteins referred to as replicase 1a and 1ab (4405 and 7096 amino acids long, respectively), which are subsequently processed into the Nsps. The remaining 10 kb of the viral genome encode the other structural proteins, namely the Spike (S), Envelope (E), Membrane (M), and Nucleocapsid (N) proteins as well as various accessory proteins (open reading frames; ORFs).^37,38^ We searched for zinc(II)-binding proteins in the SARS-CoV-2 proteome by inspecting the available experimental 3D structures and applying a previously developed bioinformatics pipeline^26^ for the prediction of the zinc(II) proteome based only on protein sequence. The positive predictive value, also called precision, of this method is between 85% and 89%.^26^

The results are summarized in Table 1, showing that about 40% of the viral proteins are potentially zinc(II) binding. In this first analysis we did not separate replicase 1a and replicase 1b into the various Nsps because this allowed us to identify potential sites that bridge different Nsps, i.e. involve donor atoms from protein residues of two distinct Nsps. In Table 1 we separated experimentally characterized zinc(II)-binding sites (i.e. sites observed in experimental 3D structures, Supplementary Table S1) from those that are only predicted (Supplementary Tables S2–S4); as a result, we identified 37 zinc(II)-binding sites, which were compared to those of closely related sequences of SARS-CoV and MERS-CoV by multiple alignment. Most of the predicted zinc(II)-binding residues are conserved in all three viral proteomes, or at least in SARS-CoV and SARS-CoV-2 (Supplementary Tables S1–S4).

**Table 1:** SARS-CoV-2 proteins containing structurally characterized (experimental) or predicted zinc(II)-binding sites

Zinc(II) protein	Uniprot Id	Experimental sites	Predicted sites
Replicase polyprotein 1a	P0DTC1	7	9
Replicase polyprotein 1b^a^	P0DTD1	8	5
Spike glycoprotein	P0DTC2	0	4
ORF3a protein	P0DTC3	0	2
ORF3d	P0DTG0	0	1
ORF8 protein	P0DTC8	0	1
Total		15	22

^a^The values in this row take into account only the portion encoded by ORF1b.

A detailed analysis of the distribution of the zinc(II)-binding sites over the individual Nsps as well as for the viral ORFs is given in Table 2. In particular, we separated predicted sites into three groups: (i) those in which it was possible to verify the spatial proximity of the residues forming the predicted sites (from AlphaFold predictions^35^ or from the analysis of the structures of similar viral proteins,^39^ Supplementary Table S2); (ii) those that do not have this kind of support (Supplementary Table S3); and (iii) the bridging sites (Supplementary Table S4), which also lack structural support. While the presence of predicted zinc(II) sites in experimental structures validates this bioinformatics approach, their absence can be attributed to several factors; e.g. the zinc(II) ion can be lost during purification or never be incorporated into the target protein. Affinity for zinc(II) varies significantly among different protein classes.^40–42^ Zinc(II) enzymes and zinc(II) structural domains that show high affinity for zinc(II) are often associated with a slow dissociation rate, thus retaining a bound zinc(II) ion during purification. Other proteins, whose function depends on the local availability of zinc(II), can be activated or inhibited only transiently. Also low affinity sites can be functionally relevant, because they respond to dynamic changes in zinc(II) and protein concentration in a specific cellular compartment and are sensitive to physiological conditions or disease states. This is particularly relevant for the viral replication niche,^43^ where host transporters and accessory factors can be recruited.^44^ Out of the 16 Nsps of SARS-CoV-2, 10 bind at least one zinc(II) ion (corresponding to about 65% of Nsps that compose replicase polyproteins). This result indicates that zinc(II)-binding sites have a role in virus replication; as described later in this paper, these sites have a high structural similarity to zinc fingers sites of human DNA/RNA-interacting proteins. Several sites are intermolecular, i.e. they bridge different Nsps. Bridging sites may have a role in tuning the structure of the replicase polyproteins before their cleavage, by modulating domain–domain interactions, or in the formation of protein complexes. In this respect, the role of zinc(II) to induce protein–protein interactions has received considerable attention and is now well established.^40^^,^^45^^,^^46^ In the InterMetalDB database, zinc(II) is the second-most common interfacial metal ion, closely following calcium(II).^47^ Intriguingly, the prediction and experimental validation of zinc(II)-driven protein–protein (either intermolecular or inter-domain) interactions is still regarded as an outstanding question in bioinorganic chemistry.^46^

**Table 2:** Distribution of zinc-(II)-binding sites for all nonstructural proteins (Nsps), the Spike protein, and zinc(II)-binding open reading frames (ORFs) of SARS-CoV-2^a^

				Predicted sites			
	Protein	Length	Experimental sites	With modeling support	Other	Bridging sites	Max
1	Nsp1	180	0	0	0	0	0
2	Nsp2	638	3	0	0	0.5	3.5
3	Nsp3 (Papain-like Protease)	1945	1	3	2	0.5	6.5
4	Nsp4	500	0	0	0	0.5	0.5
5	Nsp5 (Main protease)	306	1	1	0	0.5	2.5
6	Nsp6	290	0	0	0	0	0
7	Nsp7 (RNA polymerase)	83	0	0	0	0	0
8	Nsp8 (Primase)	198	0	0	0	0	0
9	Nsp9 (RNA-binding protein)	198	0	0	0	0	0
10	Nsp10	139	2	0	1	0	3
11	Nsp11	13	0	*Not computed*			
12	Nsp12 (RNA-polymerase)	932	2	0	1		3
13	Nsp13 (helicase)	932	3	0	1	0.5	4.5
14	Nsp14 (exoribonuclease)	527	3	0	2	0.5	5.5
15	Nsp15 (endoribonuclease)	346	0	0	0	0	0
16	Nsp16 (methyltransferase)	298	0	0	0	0	0
17	Spike glycoprotein	1273	0	0	4	0	4
18	ORF3a protein	274	0	1	1	0	2
19	ORF3d	154	0	0	1	0	1
20	ORF8 protein	121	0	0	1	0	1
	Total		15	5	14	3	37

^a^The Max column reports the sum of all experimental and predicted sites, i.e. the maximum possible number of sites for each protein. Sites predicted to bridge two different Nsps were assigned as equally split between the two (i.e. 0.5 each).

**Fig. 1:**
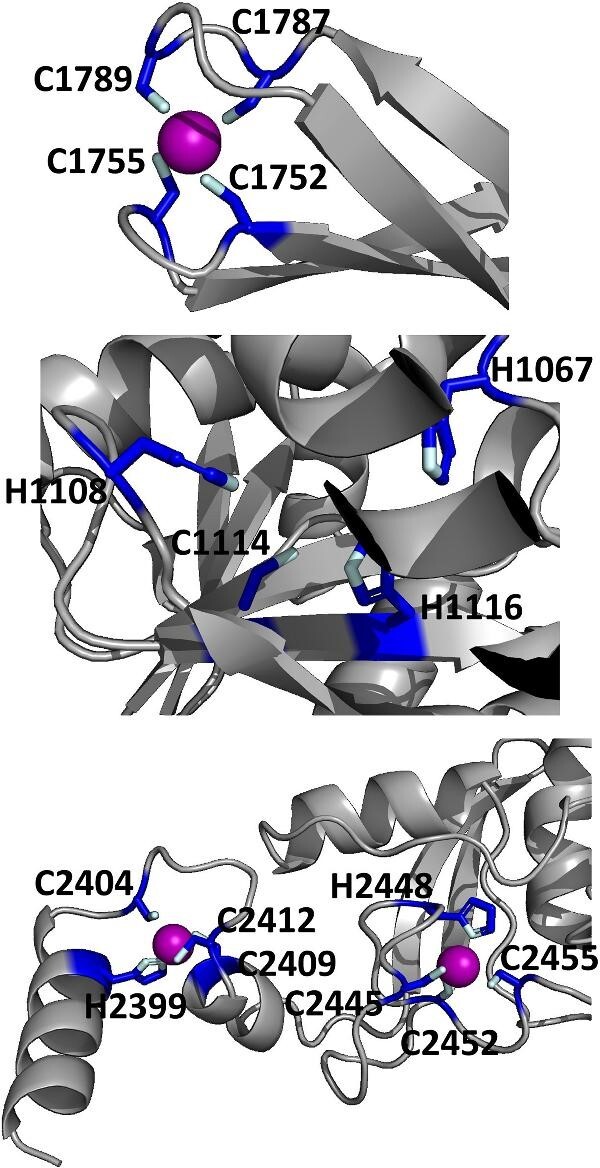
The zinc(II)-binding sites of Nsp3. Top: experimental site (PDB ID 6WRH); middle and bottom: predicted zinc(II) sites. In all panels, the zinc(II) ion is in purple, the protein donor atoms are in cyan, zinc(II) ligands are in blue.

The protein with the largest number of potential sites is Nsp3 (six sites, plus a bridging site), followed by Nsp13 (four, plus a bridging site), and then by the Spike protein (four sites). All the amino acids forming the zinc(II)-binding sites in Nsp3 are conserved in coronaviruses. Figure 1 shows the zinc(II)-binding sites in Nsp3 that have structural support. An experimentally characterized site is present in the PLP2 domain,^48^ while the other three putative sites are located, one in the Mac1 (ADP-ribose-phosphatase) domain and two in the Y1 domain. The experimentally characterized zinc(II)-binding site of Nsp3 serves a structural role for the catalytic PLP2 domain.^48^ The latter role is in agreement with the coordination environment of the zinc(II) ion, which is formed by four cysteines as commonly observed in structural zinc(II) sites.^49^ The binding site is strictly conserved in coronavirus sequences and is essential for activity.^50^ Notably, in the presence of inhibitors, PLP2 binds various additional zinc(II) ions, including one that is coordinating by the active site residues Cys111 and His272.^48^ It seems unlikely that these sites are populated by the metal during the normal replication cycle of the virus. The enzymatic activity of the Mac1 domain allows the virus to counteract the action of poly(ADP-ribose) polymerase (PARP) proteins. ADP-ribosylation is regarded as a crucial mechanism for regulation of the antiviral response of the cell.^51^ The antiviral role of human PARP proteins is linked both to their involvement in the signaling cascade of the interferon response^52^ and to their ability to interact with/modify viral RNA and proteins to suppress replication. In this context, it is quite interesting that the antiviral effect of PARP7 and PARP13 is not linked to their catalytic activity, but instead to the interaction of their zinc finger domains with the viral RNA.^53^^,^^54^ The Y1 domain is structurally and functionally uncharacterized.^55^^,^^56^ The 3Ecto domain of Nsp3 was initially proposed to harbor one or more metal-binding sites, due to the presence of a cluster of His and Cys residues.^57^ However, due to the low conservation of the cluster in different viruses this idea was subsequently abandoned,^58^ and indeed our approach did not identify any sites in the 3Ecto domain.

**Fig. 2:**
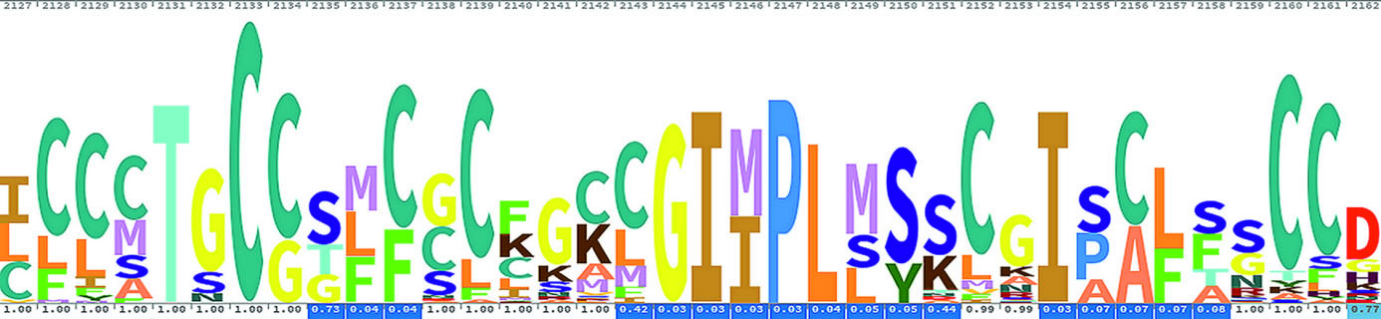
Skylign^85^ of the multiple sequence alignment of the C-terminal Cys-rich sequence of coronaviral Spike proteins. The Skylign highlights the presence, at each alignment position, of amino acids occurring more frequently than in a random distribution. The higher the letter, the more frequent is the occurrence of the corresponding amino acid at each position.

Our approach also highlighted the presence of 10 potential zinc(II)-binding cysteines in the C-terminal cytoplasmic region of the Spike protein. These cysteines, which are highly conserved in coronaviruses (Fig. 2), have a distribution along the sequence similar to that observed in metal-sequestering metallothioneins.^59^ They could thus bind a cluster of three or four zinc(II) ions. Since the Spike protein forms a trimer, a total of 9–12 zinc(II) ions could be bound to each complex. The cytoplasmic tail of the SARS-CoV-2 Spike protein facilitates the delivery of the protein to the cell surface and syncytia formation.^60^ This allows the virus to spread to neighboring cells avoiding exposure to the immune system. Some of the cysteines in the membrane-proximal half of Spike are known to be palmitoylated in SARS-CoV and other coronaviruses and, once modified, are likely to be embedded in the surface of the bilayer, thus facilitating cell fusion.^61^ Proteins of the palmitoyl acyltransferase family contain an Asp–His–His–Cys zinc finger domain whose inhibition reduces Spike palmitoylation. Interestingly, a diverse family of enveloped RNA viruses, arenaviruses, possess a cytoplasmic domain in the fusion subunit of the glycoprotein complex with two distinct clusters of Cys and His residues posed to coordinate two zinc(II) ions, which are involved in the mechanism of membrane fusion and virus entry into host cells.^62^

**Fig. 3:**
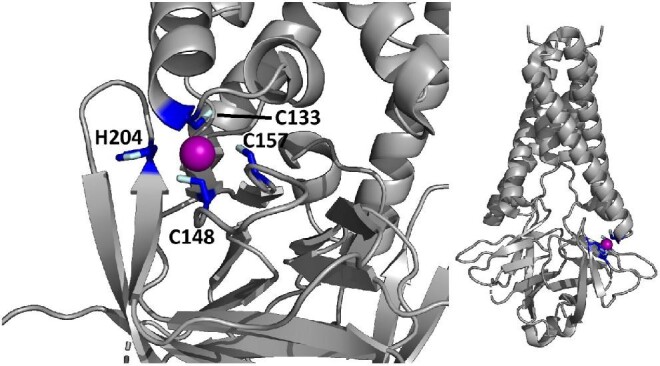
The predicted zinc(II)-binding site of ORF3a. The color code is as in Fig. 1.

The experimental cryoEM structure of ORF3a does not contain any metal ions.^63^ The metal site that we predicted is located within a region described as a cysteine-rich patch,^63^ with the addition of the nearby His204 (Fig. 3). This region is adjacent to the tunnels connecting the core part of the structure to the cytosol. While the distances among the cysteines are too large to allow formation of disulfide bridges in the absence of significant structural rearrangements,^63^ a relatively small-scale reorientation of the side chains would yield a properly formed zinc(II)-binding site. Thus, it is possible that this predicted site indeed has a physiological role, such as regulating the activity of Orf3a, which is a putative ion channel (a viroporin),^64^ in a concentration-dependent manner.

Regarding ORF8, the possibility that it binds zinc(II) has been mentioned in previous works,^65^^,^^66^ along with ORF7. The latter interaction has received some experimental support recently,^66^ while no experiments could be performed for ORF8 due to insufficient sample availability. Our predictions did not identify a potential zinc(II)-binding site in the sequence of ORF7.

### Search for local structure similarities of SARS-CoV-2 zinc(II)-binding sites to other zinc(II)-binding sites in the PDB

We investigated whether the zinc(II)-binding sites of SARS-CoV-2 proteins bear any structural resemblance to the zinc(II)-binding sites of other proteins. To this end, we compared the local structures around the metal sites, both experimental and based on computational models, to the content of the MetalPDB database of macromolecular metal-binding sites,^30^ using the MetalS^3^ search tool.^36^ This tool only compares the structural environment around the metal ion, regardless of the entire protein fold, allowing the detection of distant similarities even across distinct protein families,^27^^,^^67^ and therefore revealing potential functional similarities,^68^ as well as possible competing sites. All the structurally similar sites retrieved by our search (Table 3) contained zinc(II). In the large majority of cases, the top-scoring hit was a human (for five viral sites) or another eukaryotic protein (for four viral sites). Two distinct eukaryotic proteins showed similarity to the structural site of the PLP2 domain of Nsp3. Thus, eight distinct viral zinc(II)-binding sites bear resemblance to eukaryotic sites. Notably this search included the presently predicted viral sites with structural support, as we could build models of the zinc(II)-occupied structure. In particular, one of the predicted sites in the Y1 domain of Nsp3 bears significant resemblance to the zinc(II) site of CCAAT/enhancer-binding protein (C/EBP) epsilon, a human nuclear protein that binds DNA. Another intriguing similarity was detected between one of the three sites of Nsp2 and the single zinc(II)-binding site of the yeast mitochondrial import protein ZIM17 (PDB ID 2E2Z.^69^) The zinc finger of Zim17 is important for protein translocation into the matrix, by binding to unfolded proteins in cooperation with mtHsp70.^70^ The human homolog of Zim17 is Hep1 (also called DNLZ), whose likely function is to act as a co-chaperone toward mortalin (i.e. mammalian mtHsp70).^71^ The structure of Hep1 is indeed stabilized by zinc(II). SARS-CoV Nsp2 is known to interact with prohibitin 1 and 2 (PHB1 and PHB2, respectively),^72^ which are involved in the maintenance of mitochondrial integrity.^73^ These data altogether suggest that Nsp2 could play multiple roles in the impairment of mitochondrial function caused by SARS-CoV-2, by interfering with the import of mitochondrial proteins as well as with mitochondrial stabilization mechanisms.

**Table 3:** Structural similarity between SARS-CoV-2 and other zinc(II)-binding sites^a^

Viral protein	Zinc(II)-binding residues in viral protein (numbers refer to Uniprot P0DTD1)	Structurally similar site in MetalPDB	Organism of the MetalPDB site	Protein harboring the MetalPDB site (Uniprot ID)	Structural superposition
Nsp2	C323, C326, C341, C344	2e2z_1	*Saccharomyces cerevisiae* ^b^	Mitochondrial protein import protein ZIM17 (P42844)	
					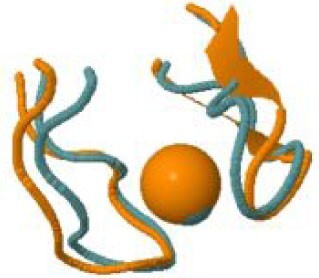
Nsp3	C1752, C1755, C1787, C1789 (PLpro)	5vjj_6	*Melampsora lini*	Avirulence protein AvrP123 (B2ZCS6)	
					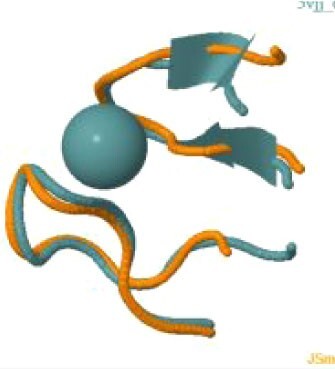
Nsp3	C1752, C1755, C1787, C1789 (PLpro)	2jr7_1	*Homo sapiens*	DPH3 homolog (Q96FX2)	
					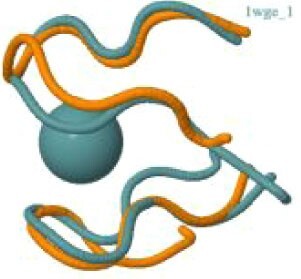
Nsp3	H2399, C2404, C2409, C2412	3t92_3	*Homo sapiens*	CCAAT/enhancer-binding protein epsilon (Q15744)	
					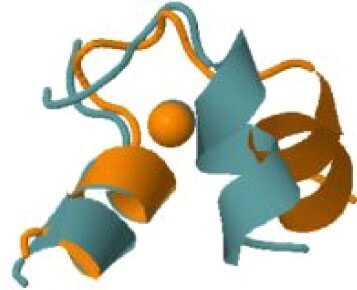
Nsp10	C4327, C4330, C4336, C4343	3mhs_2	*Saccharomyces cerevisiae* ^b^	Ubiquitin carboxyl-terminal hydrolase 8 (P50102)	
					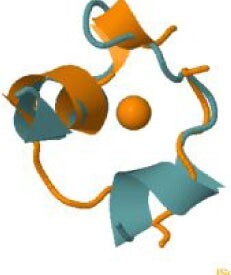
Nsp10	C4370, C4373, C4381, C4383	2ctu_1	*Homo sapiens*	Zinc finger protein 483 (Q8TF39)	
					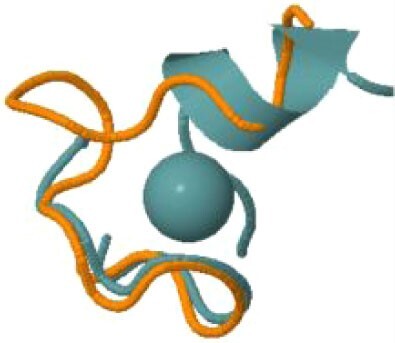
Nsp13	C5329, H5332, C5350, C5353	5e6c_2	*Homo sapiens*	Glucocorticoid receptor (P04150)	
					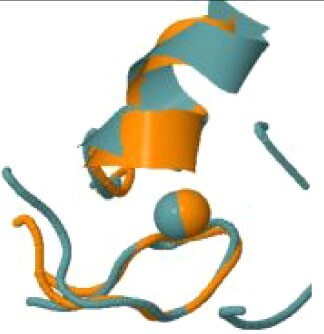
Nsp13	C5340, C5343, H5357, H5363	2xzl_4	*Saccharomyces cerevisiae* ^b^	ATP-dependent helicase NAM7 (P30771)	
					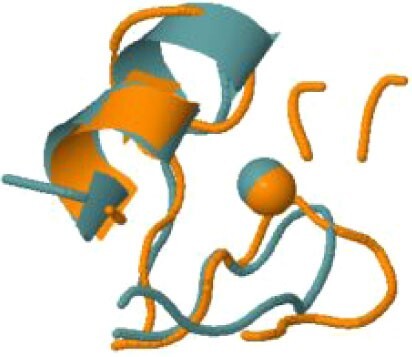
Nsp13	C5374, C5379, C5396, H5399	5dah_3	*Homo sapiens*	Protein AF-10 (P55197)	
					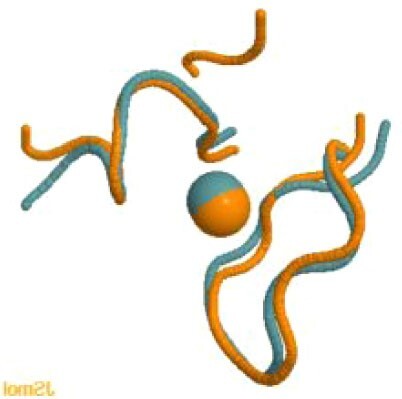
Nsp14	C6132, C6135, C6151, H6154	2naa_2	*Mus musculus* ^b^	Histone-lysine N-methyltransferase, H3 lysine-36 specific (O88491)	
					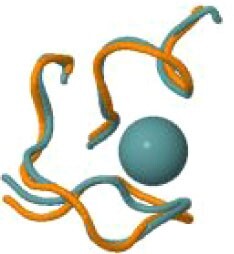
Nsp14	H6182, C6186, H6189, C6204	5bqk_3	*Human herpesvirus 1*	mRNA export factor (P10238)	
					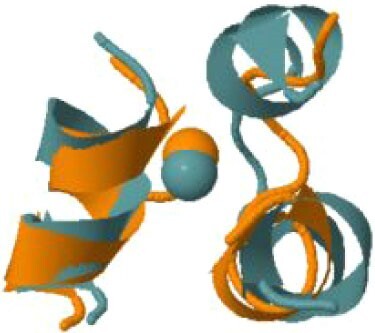

^a^Only significant hits retrieved by MetalS^3^ in the MetalPDB database are shown. The structural comparison is limited to the metal site and does not involve the overall protein fold.

^b^There is a human homologue with a conserved site but no experimental 3D structure.

We observed that the majority of structurally similar eukaryotic sites are zinc fingers, several of which had already been detected in SARS-CoV-2 protein structures.^74^ Instead, zinc fingers are quite uncommon in bacterial pathogens.^75^ In the absence of experimental data on the zinc(II)-binding constants of viral proteins, some hints can be derived from the knowledge of classical and nonclassical eukaryotic zinc fingers. The affinity of zinc fingers varies over nearly seven orders of magnitude (10^8^–10^15^) at pH 7 and is highly dependent on subtle changes in protein sequence and second sphere interactions (H-bond network, packing of hydrophobic residues, etc.).^40–42^ Despite this high diversity in zinc(II) affinity, which can also be reflected in the viral zinc(II)-binding sites, it is still possible to draw some similarities between the host and viral zinc finger proteins. While some catalytic and structural sites can stably bind zinc(II) ions, others can be occupied only transiently, depending on the levels of free zinc concentration, which in turn depend on the cellular conditions and subcellular localization.^76^ Zinc(II)-regulated proteins generally exhibit lower affinity for zinc(II), facilitating reversible binding. On the other hand, thermodynamically stable zinc(II) proteins may become kinetically labile in a competitive environment and directly transfer the metal ion via intermediate ternary complex formation rather than via an association–dissociation mechanism.^77^ The labile zinc fingers of PLpro (Nsp3), Nsp10, and Nsp13 are targeted and disrupted by various zinc(II) ejectors, such as the metal chelator disulfiram and the organoselenium drug ebselen.^78^ The significant similarity observed here suggests that the zinc(II)-binding proteins of the virus and the host cell may effectively compete for the intracellular zinc(II) pool during viral replication (see also next section) and/or that the virulence of coronavirus infections is also linked to the ability of viral proteins to interfere with the recognition processes of host cells. Although numerous aspects of zinc(II) transport and trafficking have been well characterized, the mechanisms for allocating zinc(II) ions within individual proteins remain largely unexplored. The recent discovery of the first zinc(II) metallochaperone, ZNG1, indicates that an interdigitated Cys6His2 zinc finger motif on the target enzyme, coordinating two zinc(II) ions with high (pM) affinity, provides the binding interface for the metallochaperone. The latter then uses Guanosine-5'-triphosphate (GTP) hydrolysis to deliver a zinc(II) ion to a catalytic site on the target enzyme having lower (nM) affinity.^79^ An interdigitated pattern of two zinc(II) ions is present in at least one SARS-CoV-2 protein, the Nsp13 helicase, and it may be speculated that the recruitment of a host metallochaperone can restore enzymatic activity under conditions of low cellular zinc(II).

### Quantification of zinc(II) requirement for SARS-CoV-2

The data of Table 2 enable a rough estimate of the number of zinc(II) ions required during the viral replication for the correct maturation of all its proteins. This estimate is based on the maximum number of sites and the count of the virions present in the infected human host.^80^ The latter ranges between 10^9^ and 10^11^. Clearly, not all proteins, particularly the Nsps, will be present in the virion at any given moment, and the number of infectious units is about four orders of magnitude smaller. Likewise, not all proteins will be produced to the same extent, with the nucleocapsid protein, which does not bind zinc(II) ions, being by far the most abundant.^81^ In practice, by multiplying the maximum number of zinc(II) sites by the highest estimate of the number of virions, we obtain an upper limit for the total amount of zinc(II) that may be required from the host by SARS-CoV-2, which is in the order of 10^–9^ g. This quantity is extremely small with respect to the total zinc(II) content in the human body, which is approximately 1–2 g. Although there are essentially no free zinc(II) ions available in the cell,^45^^,^^82^^,^^83^ our analysis indicates that the viral zinc(II)-binding sites should be able to compete with the host proteins to obtain their metal cofactor. Notably, the so-called labile zinc(II) pool [i.e. the fraction of zinc(II) ions that can be readily removed from their intracellular complexes] is not evenly distributed in all tissues and is actually more abundant in epithelial cells.^84^

## Conclusions

Our data, together with previously published structural data, show that SARS-CoV-2 proteins contain several zinc(II)-binding sites. The majority of these sites are located within Nsps, hence it is during viral replication that they are present in the host cell and need to be populated. Owing to their structural similarity, it seems likely that viral and eukaryotic sites (typically zinc fingers) have a comparable range of affinities for zinc(II) ions. Therefore, the viral proteins should be able successfully compete with human zinc(II) proteins for ions in the labile zinc pool. The total quantity of zinc(II) required in infected cells is also compatible with metal availability not being a limiting factor. By looking at the entire sequence of SARS-CoV-2 polyproteins, we observed that several predicted sites bridge (i.e. involve protein ligands from) different Nsps. Although it is not possible to build 3D structural models of such sites, this finding suggests the intriguing idea that zinc(II) ions may contribute to tuning the structural features of the polyproteins, which might ultimately affect the viral replication process.

## Supplementary Material

mfac047_Supplemental_File

## Data Availability

The data underlying this article are available in the article and in its online supplementary material.
