# Supplementary material for: The zinc proteome of SARS-CoV-2
Source: Metallomics. 2022 Jun 29;14(7):mfac047. doi: 10.1093/mtomcs/mfac047 (PMC9314716; doi:10.1093/mtomcs/mfac047)
Supplement: mfac047_Supplemental_File [file mfac047_supplemental_file.docx]

**The Zinc Proteome of SARS-CoV-2**

Claudia Andreini^1,2^, Fabio Arnesano^3,*^, Antonio Rosato^1,2,*^

^1^ Consorzio Interuniversitario di Risonanze Magnetiche di Metallo Proteine, Via Luigi Sacconi 6, 50019 Sesto Fiorentino, Italy.

^2^ Department of Chemistry and Magnetic Resonance Center (CERM), University of Florence, Via Luigi Sacconi 6, 50019 Sesto Fiorentino, Italy.

^3^ Department of Chemistry, University of Bari "Aldo Moro", Via Orabona 4, 70125 Bari, Italy

**Supplementary Tables**

**Supplementary Table S1.** **Experimental sites.** The column “Site in the sequence” refers to the numbering in the sequence of the entire polyprotein. Highlighted residues in the last column are shared among Coronavirus proteins.

| **Protein** | **Site in the sequence** | **PDB entry** | **Site in the structure** | **Site alignment with MERS and Sars-Cov proteins** |
| --- | --- | --- | --- | --- |
| Nsp2 | C200, C231, H234, H236 | 7exm | C20, C51, H54, H56 |  |
| Nsp2 | C323, C326, C341, C344 | 7exm | C143, C146, C161, C164 |  |
| Nsp2 | C370, C373, H382, C416 | 7exm | C190, C193, H202, C236 |  |
| Nsp3 | C1752, C1755, C1787, C1789 (PL-PRO) | 6wrh | C189, C192, C224, C226 |  |
| Nsp5 | H3304, H3427, D3450 | 5eu8 | H41, H164, D187 |  |
| Nsp10 | C4327, C4330, C4336, C4343 | 7jpe | C74, C77, H83, C90 |  |
| Nsp10 | C4370, C4373, C4381, C4383 | 7jpe | C117, C120, C128, C130 |  |
| Nsp12 | H4687, C4693, C4698, C4702 | 7bv1 | H295, C301, C306, C310 |  |
| Nsp12 | C4879, H5034, C5037, C5038 | 7bv1 | C487, H642, C645, C646 |  |
| Nsp13 | C5329, H5332, C5350, C5353 | 6zsl | C5, C8, C26, C29 |  |
| Nsp13 | C5340, C5343, H5357, H5363 | 6zsl | C16, C19, H33, H39 |  |
| Nsp13 | C5374, C5379, C5396, H5399 | 6zsl | C50, C55, C72, H75 |  |
| Nsp14 | C6132, C6135, C6151, H6154 | 5c8s | C207, C210, C226, H229 |  |
| Nsp14 | H6182, C6186, H6189, C6204 | 5c8s | H257, C261, H264, C279 |  |
| Nsp14 | C6377, C6402, C6409, H6412 | 5c8s | C452, C477, C484, H487 |  |

**Supplementary Table S2:** **Predicted sites with modelling support.** The column “Site in the sequence” refers to the numbering in the sequence of the entire polyprotein. Highlighted residues in the last column are shared among Coronavirus proteins.

| **Protein** | **Site in the sequence** | **3D model /structure** | **Site in the model** | **Site alignment with MERS and Sars-Cov proteins** |
| --- | --- | --- | --- | --- |
| Nsp3 | H2399, C2404, C2409, C2412 | model | H1581, C1586, C1591, C1594 |  |
| Nsp3 | C2445, H2448, C2452, C2455 | model | C1627, H1630, C1634, C1637 |  |
| Nsp3 | H1067, H1108, C1114, H1116 | 6vxs | H45, H86, C92, H94 | 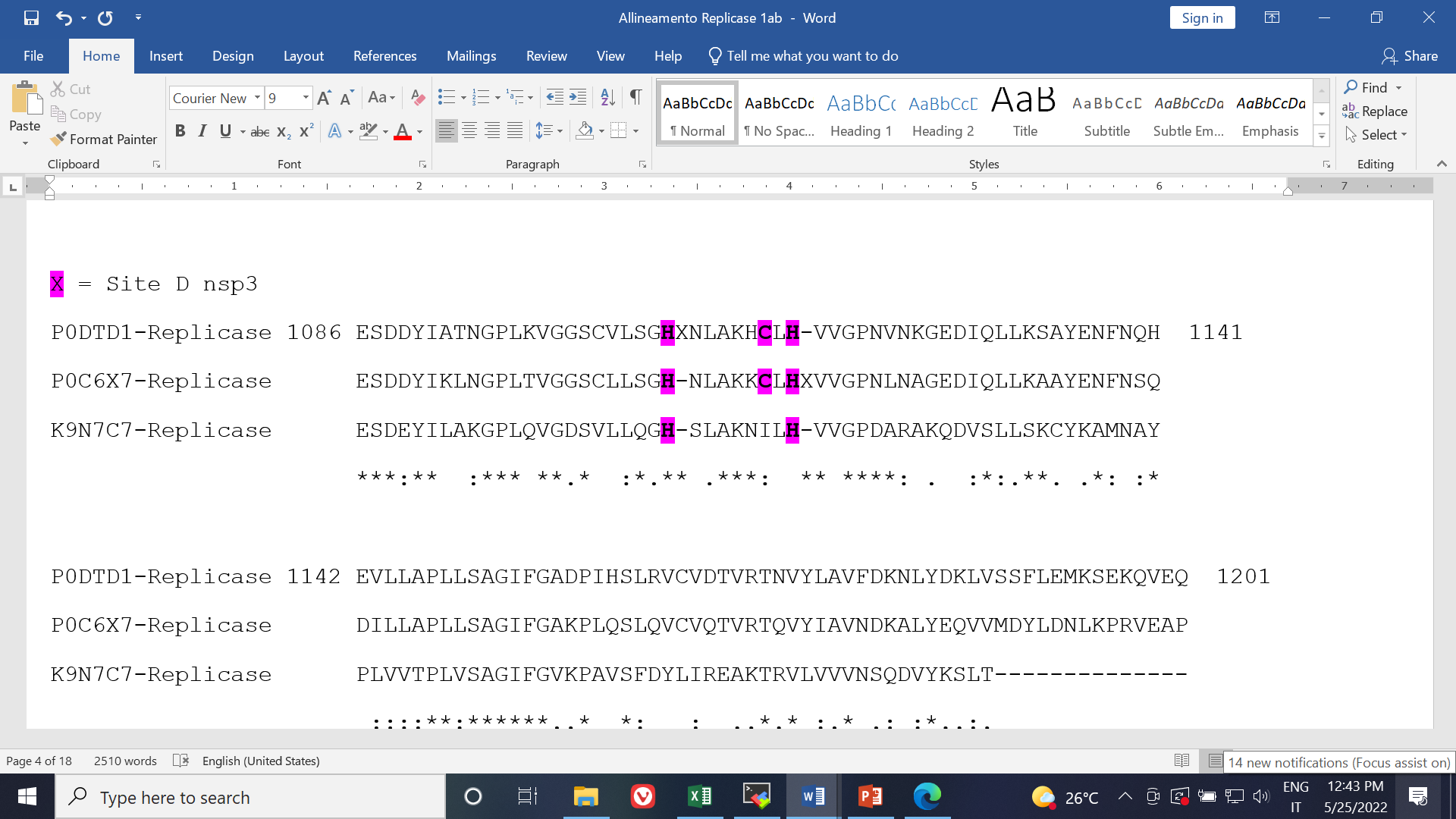 |
| Nsp5 | H3426, E3428, H3435 | 6zrt | H163, E166, H172 | 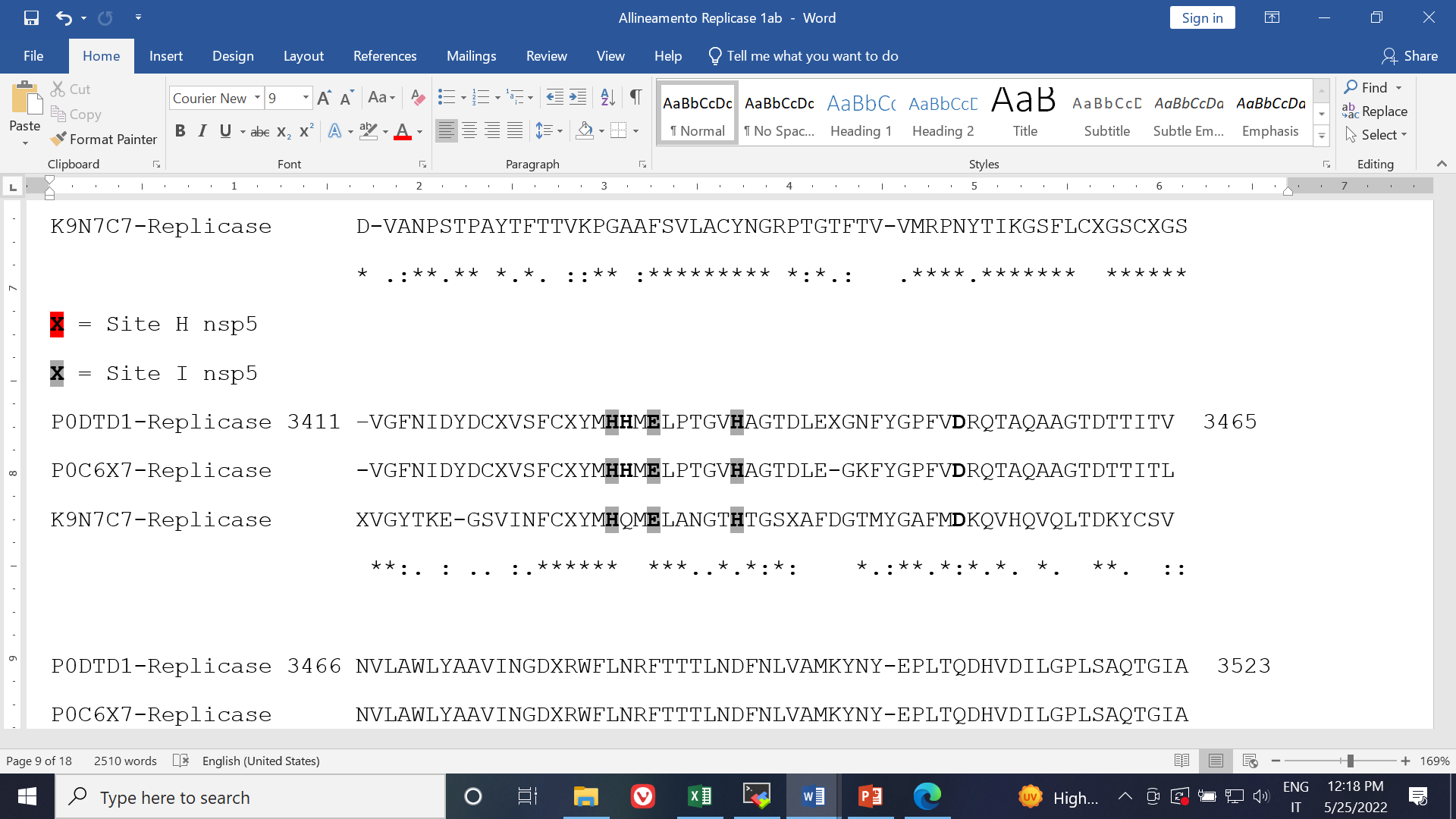 |
| Orf3a | C133, C148, C157, H204 | 6xdc | C133, C148, C157, H204 | 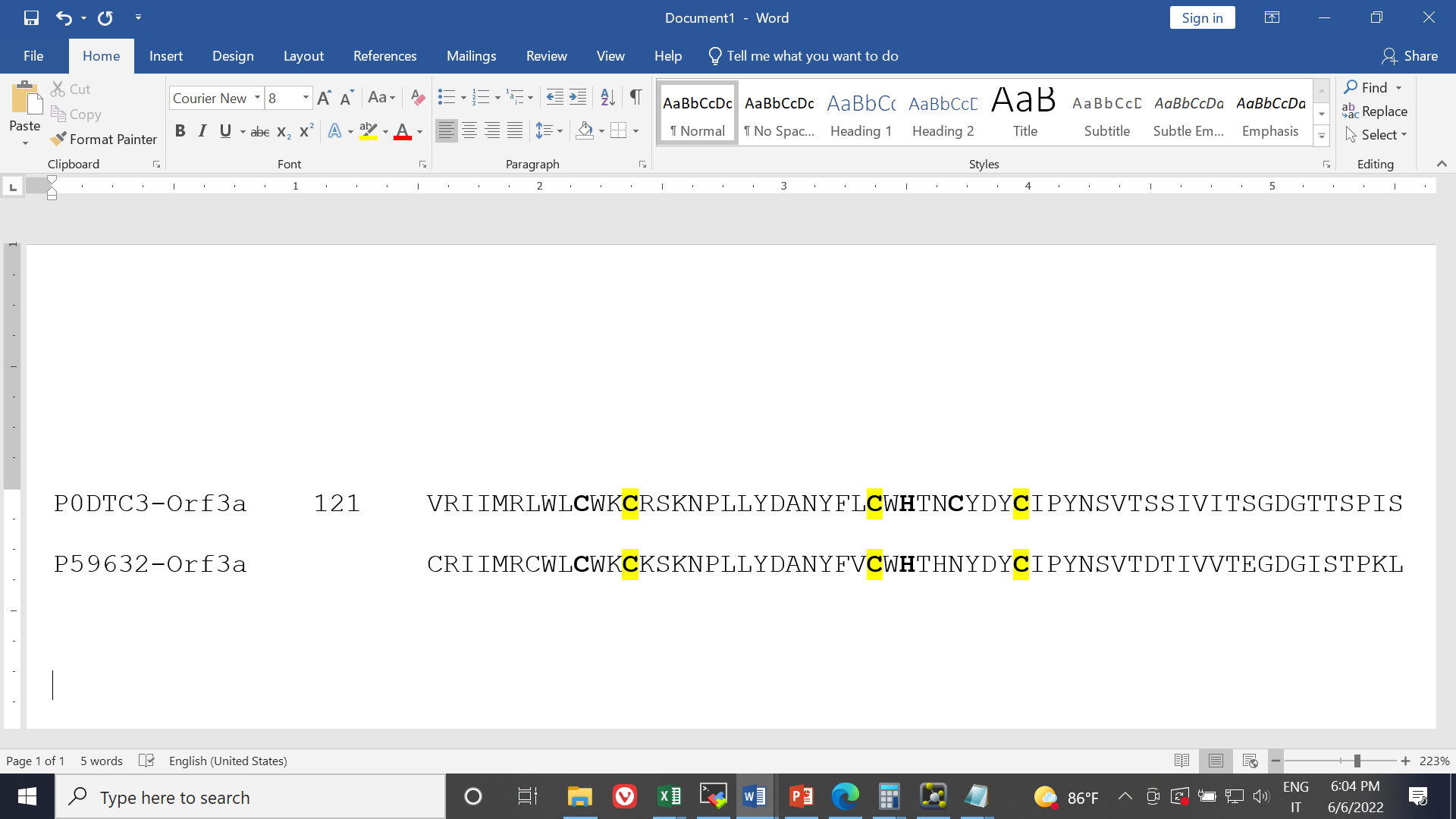  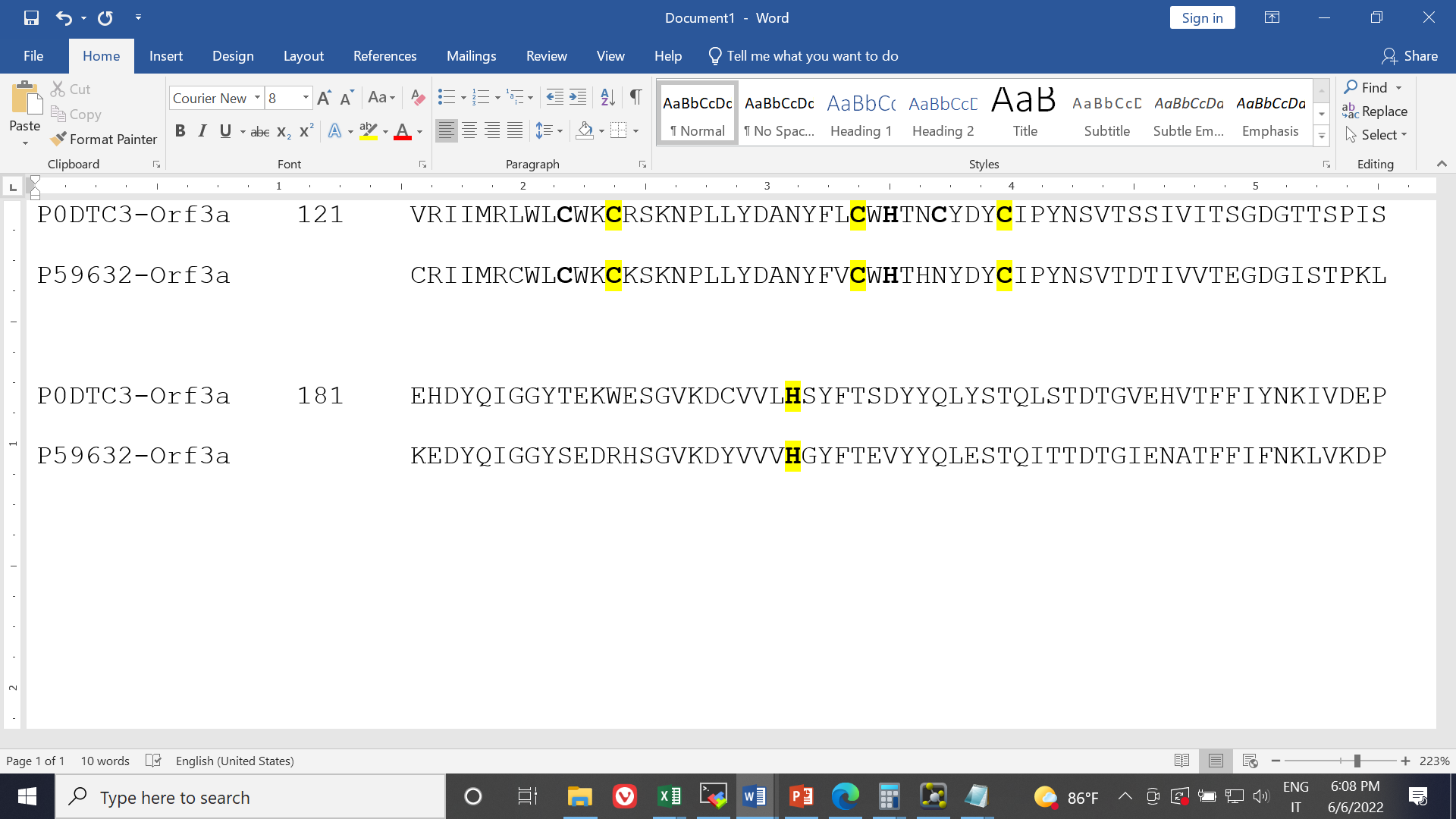 |

**Supplementary Table S3:** **Predicted sites without 3D modelling support**. Residues in bold are common to experimentally observed sites (Table S1). The highlighted residues in the last column are shared among Coronavirus proteins.

| **Protein** | **Predicted site** | **Site alignment with MERS and Sars-Cov proteins** |
| --- | --- | --- |
| Nsp3 | H1264, D1282, H2520 | 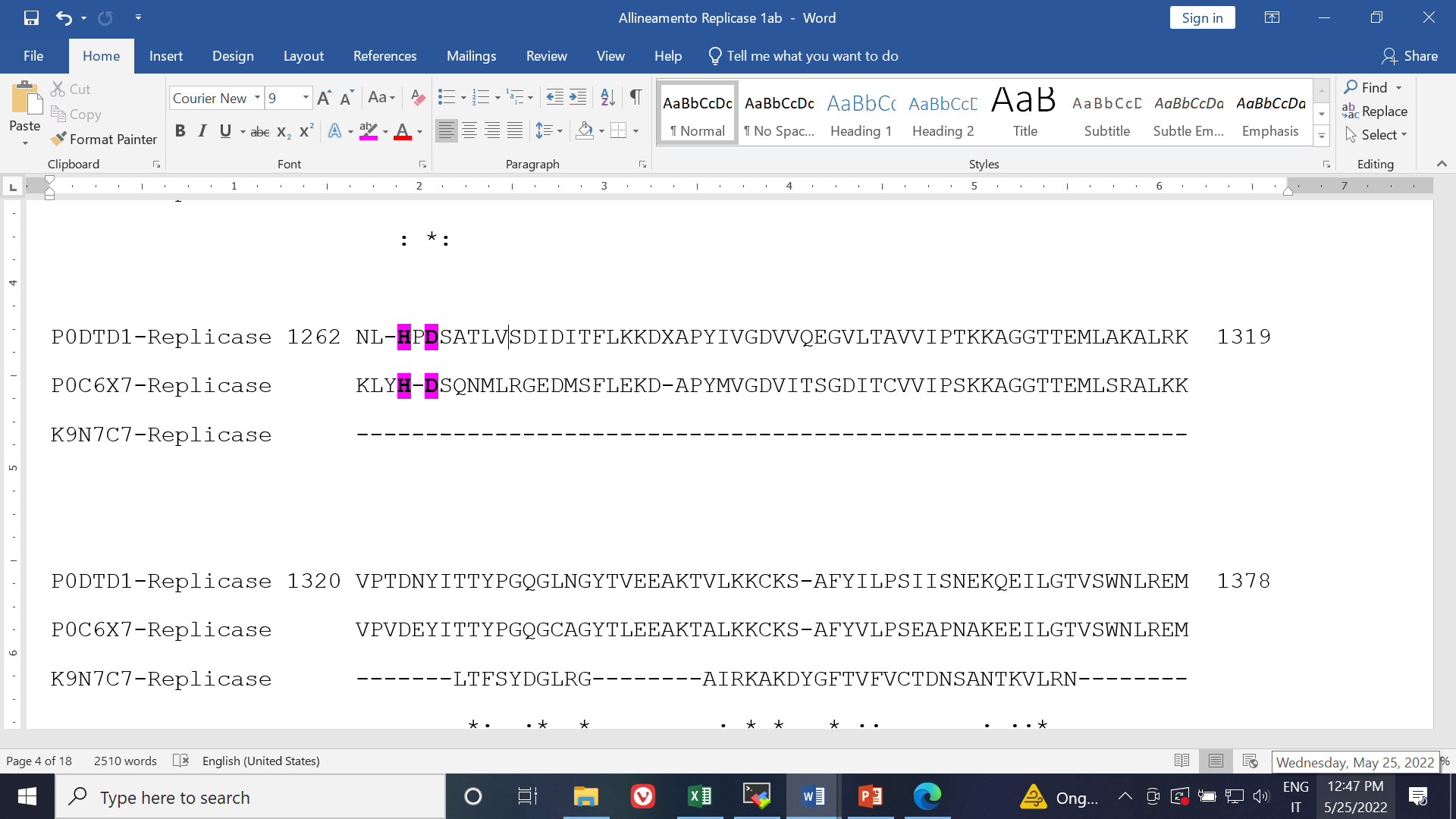  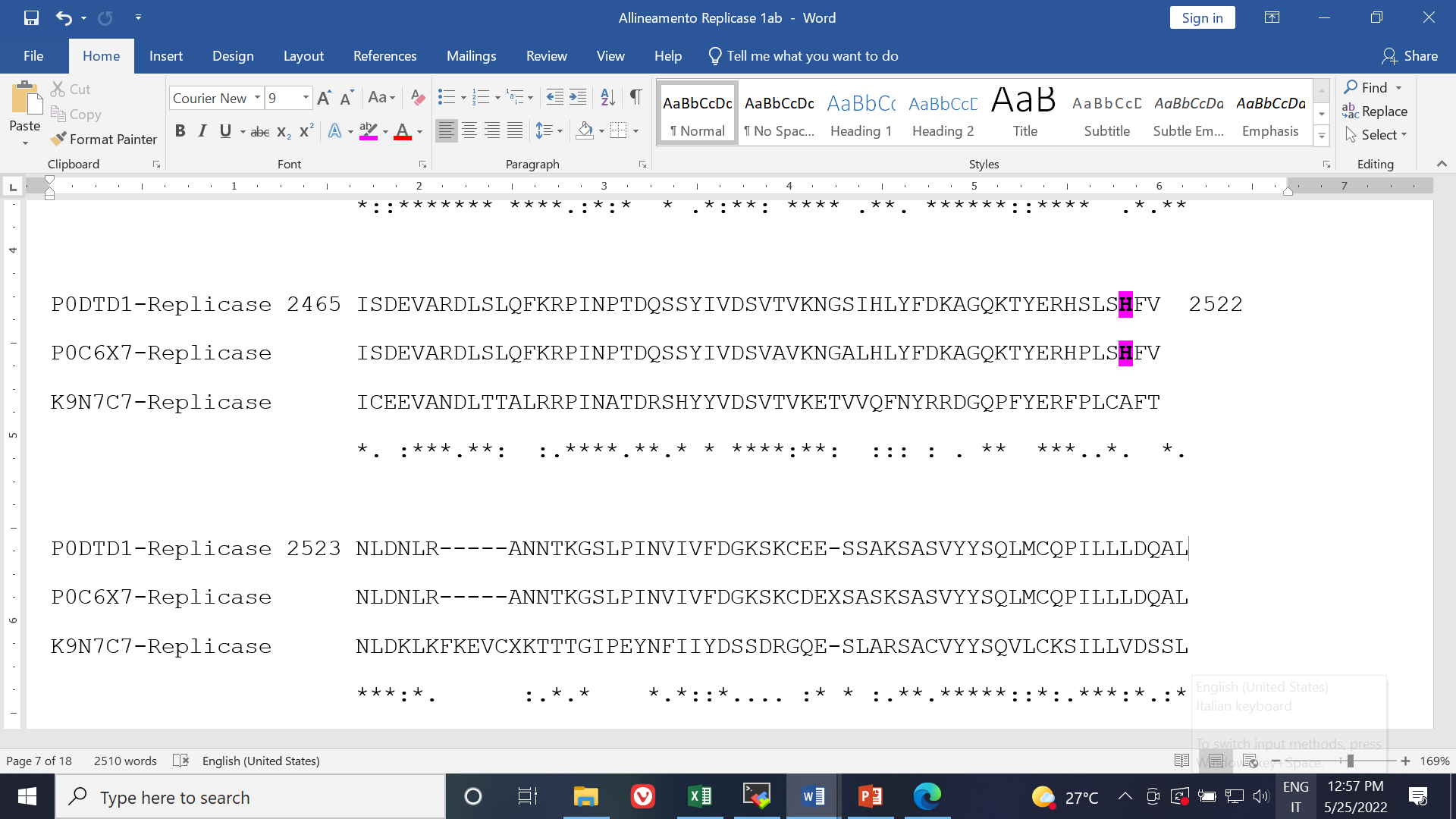 |
| Nsp3 | C2263, C2282, C2288, C2291 | 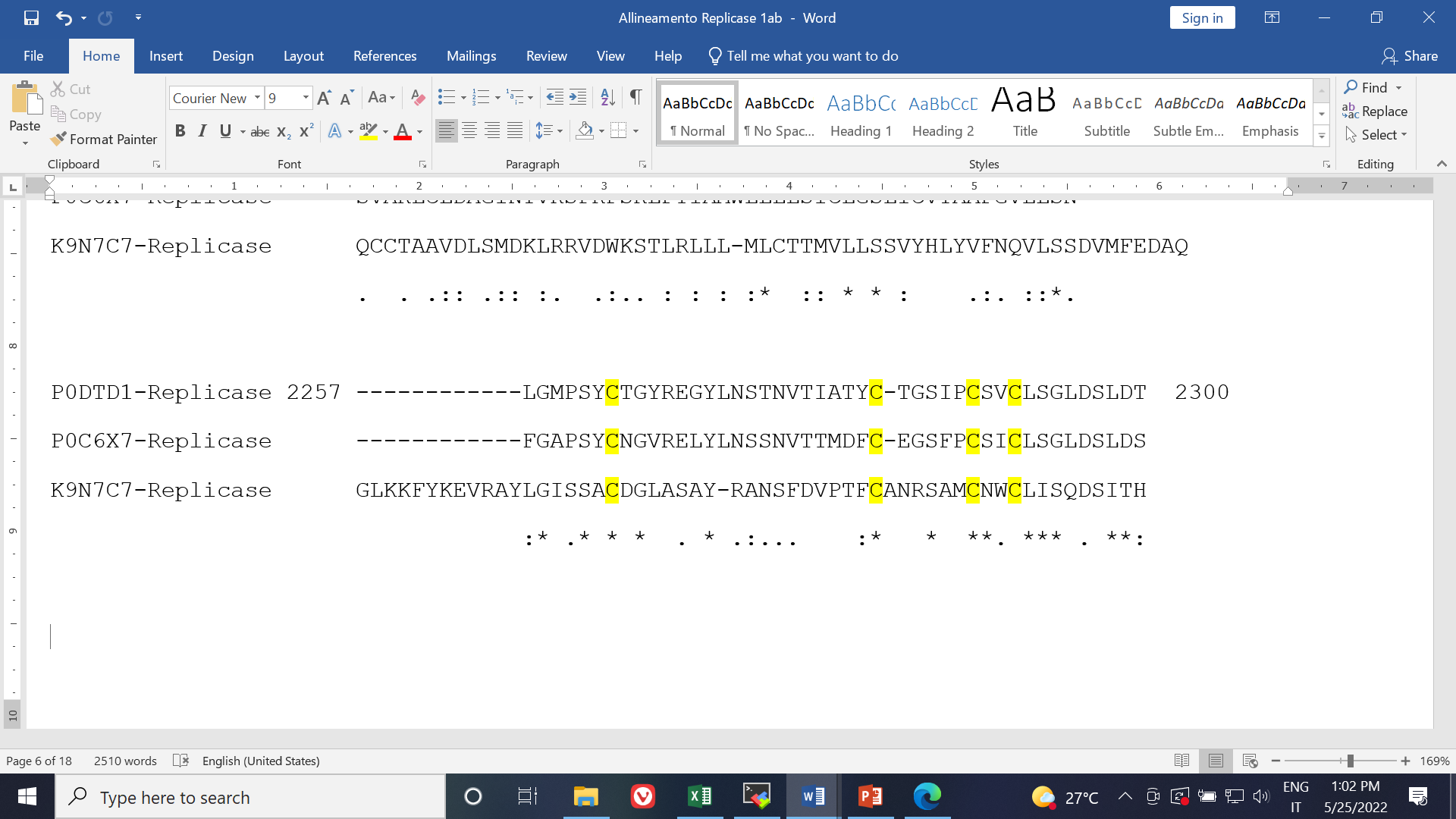 |
| Nsp10 | C4299, H4301, C4326, **C4330** | 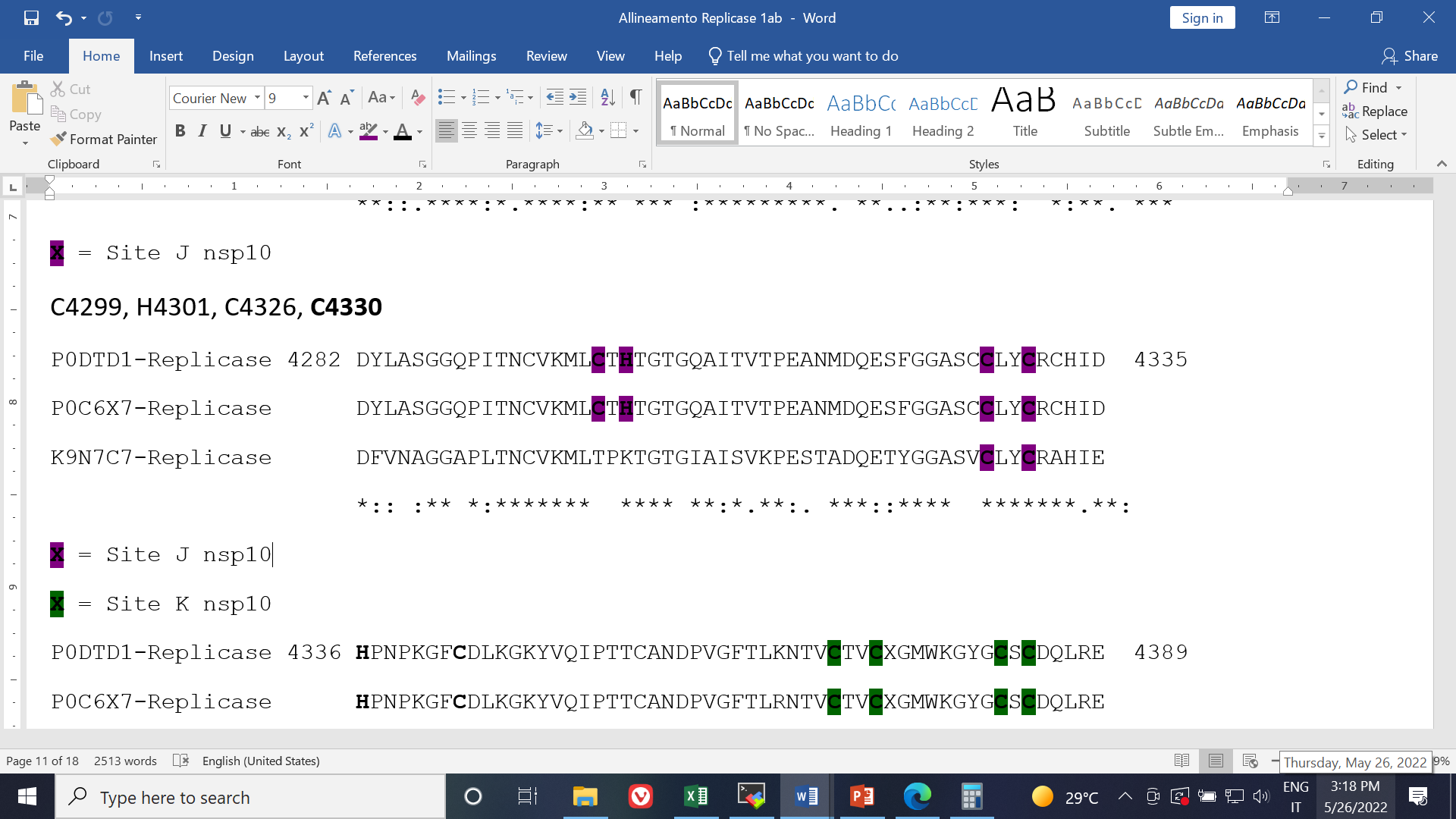 |
| Nsp12 | C4690, **C4693, C4698,** H4701 | 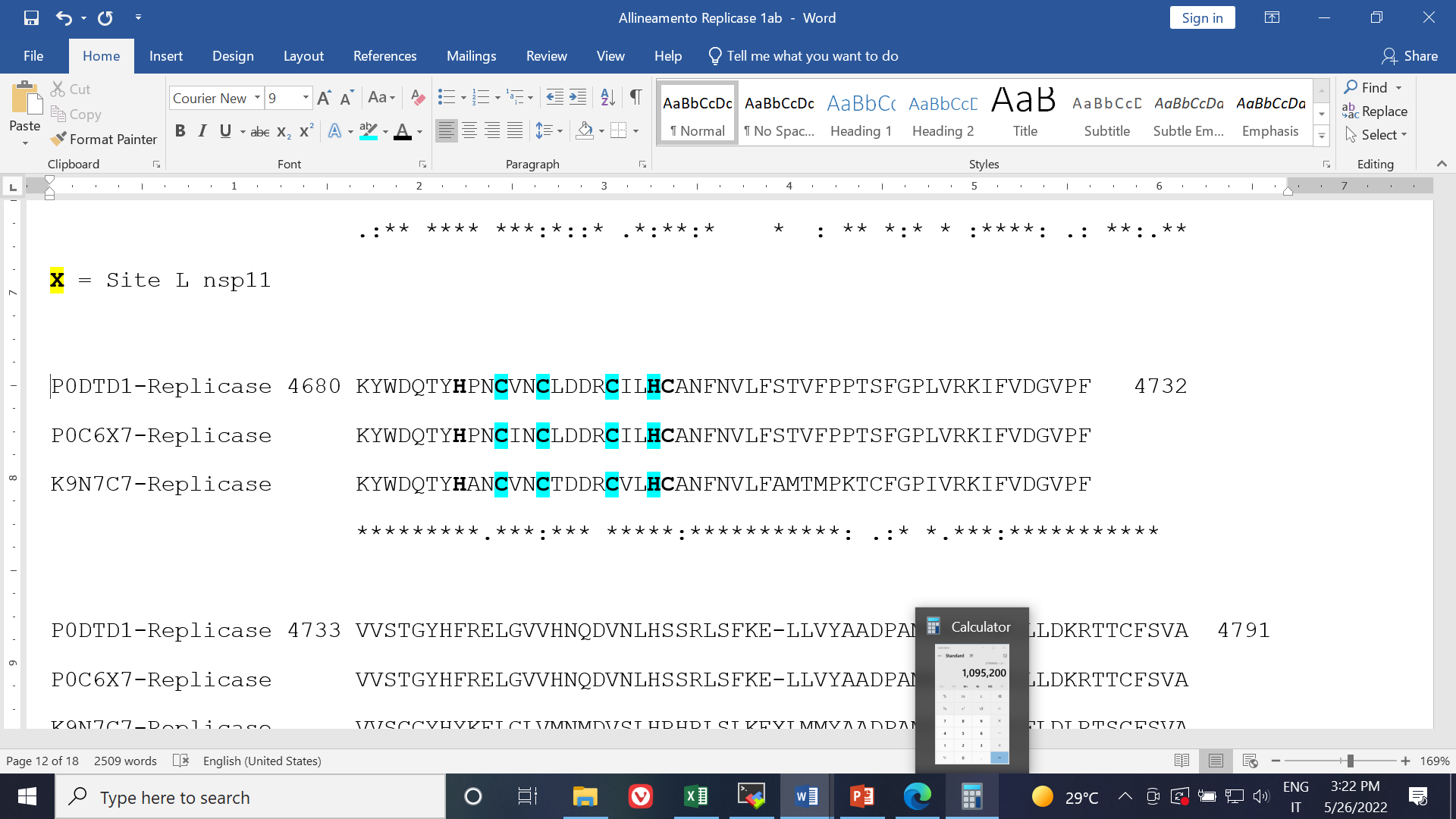 |
| Nsp13 | **C5329, C5332,** C5351, C5354 | 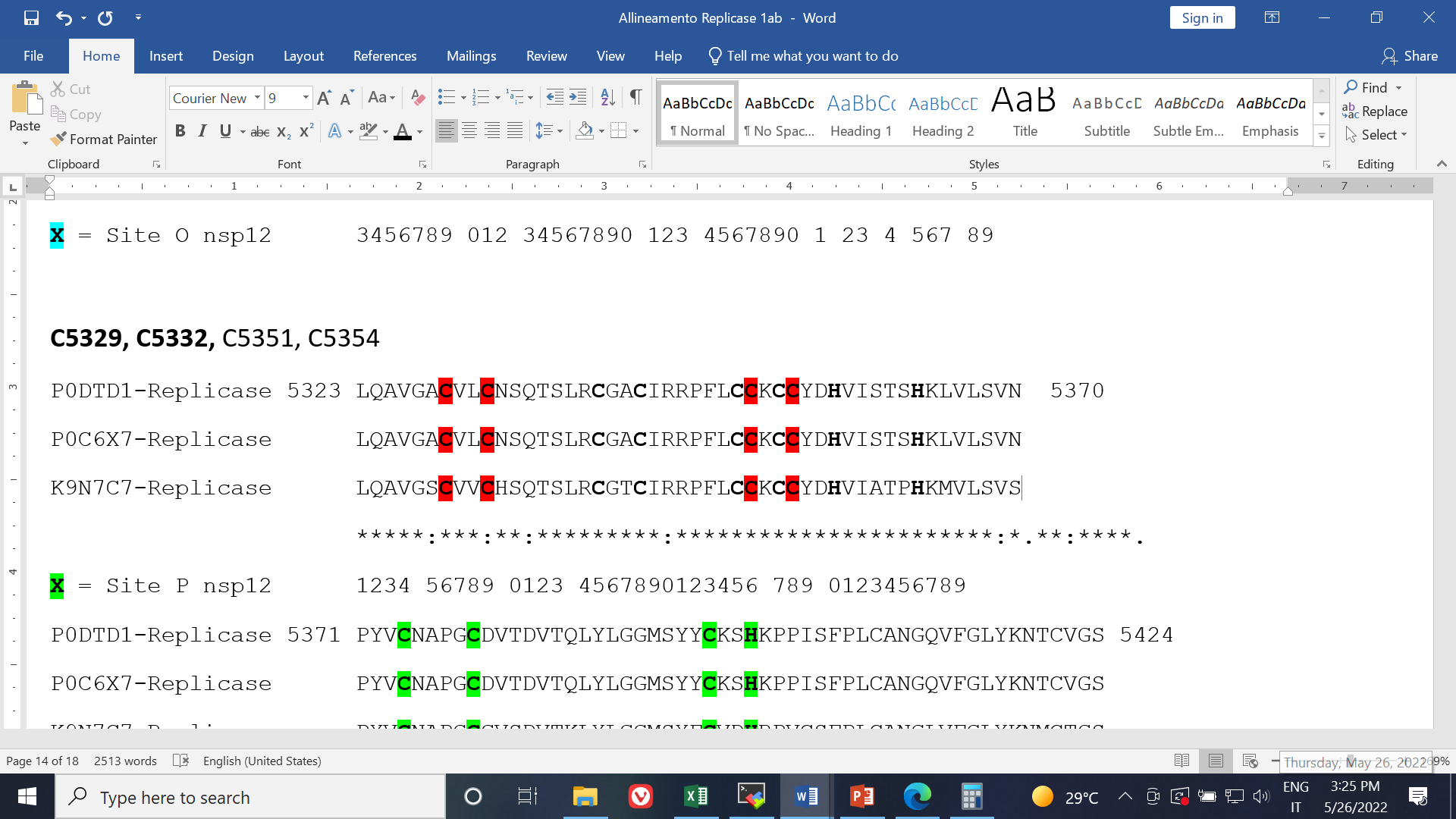 |
| Nsp14 | C6133, **C6135, C6151, H6154** | 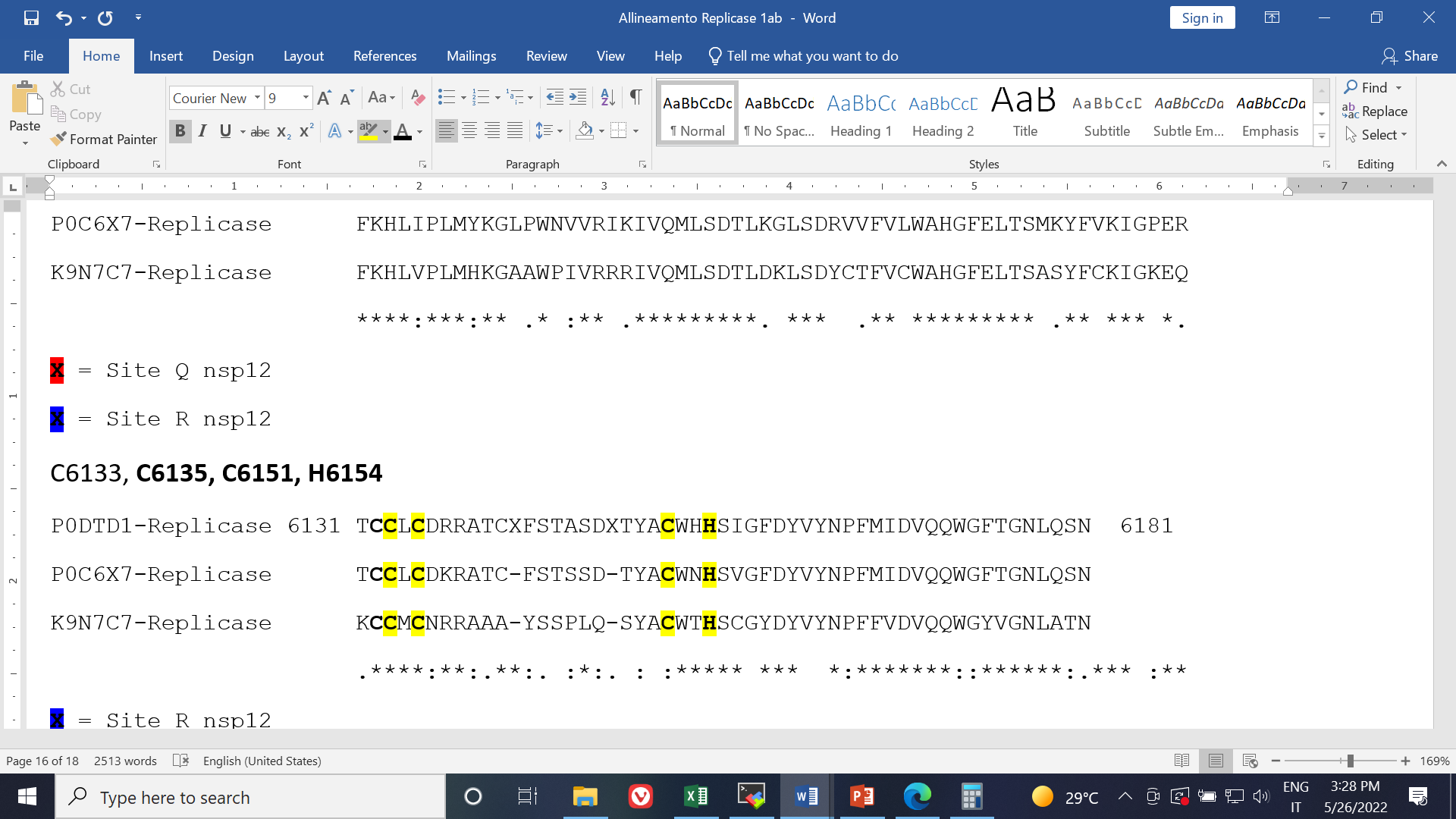 |
| Nsp14 | **C6186, H6189, C6204,** C6210 | 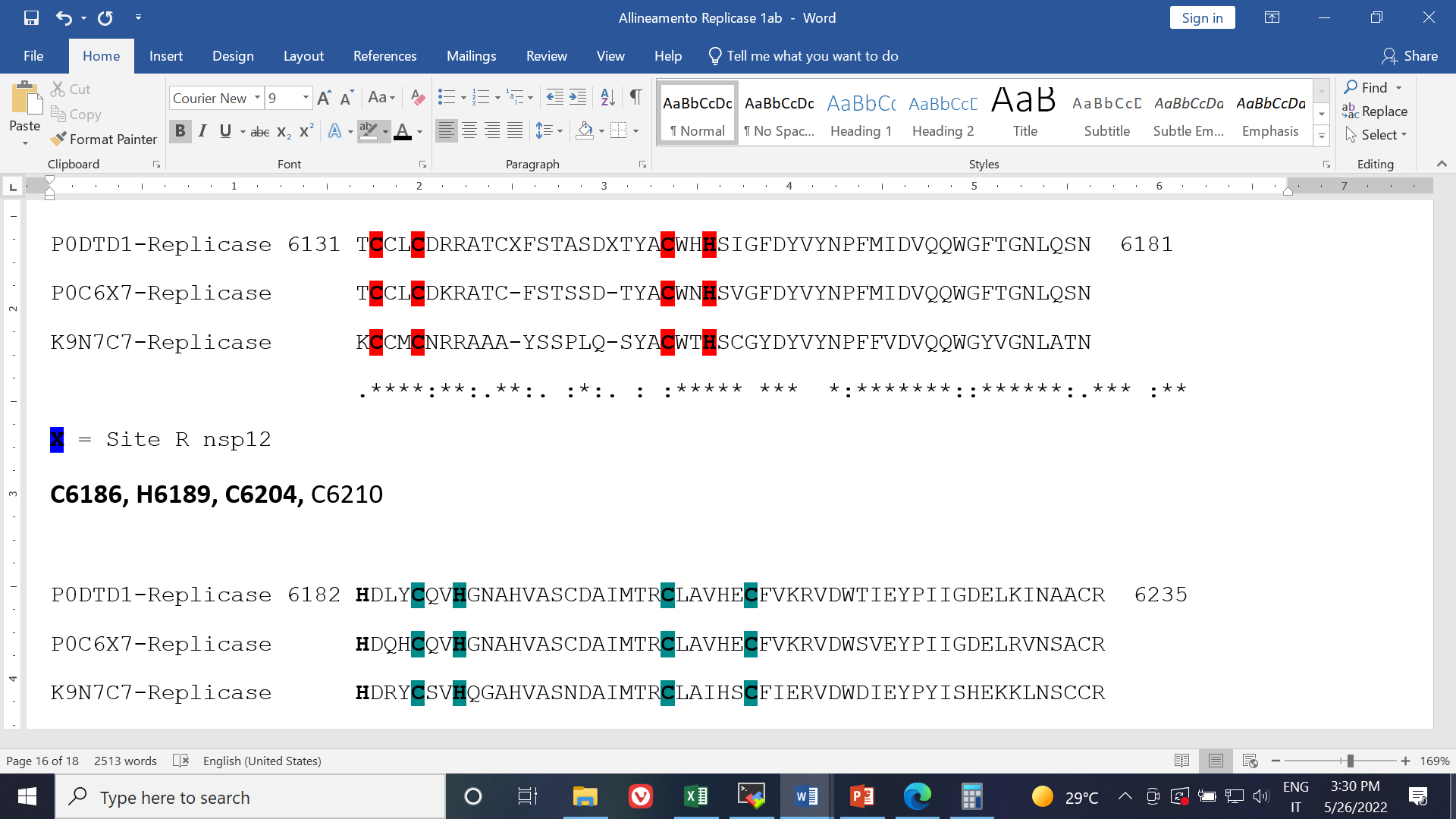 |
| Spike | C1240, C1243, C1248, C1253 |  |
| Spike | C1241, C1243, C1247, C1254 |  |
| Spike | C1241, C1243, C1248, C1253 |  |
| Spike | C1240, C1243, C1250, C1253 |  |
| Orf3a | C130, **C133**, H150, C153 |  |
| Orf3d | C4,C7,C33 | No homologs found in Sars-CoV and MERS |
| Orf8 | H17, C20, C25, H28 | No homologs found in Sars-CoV and MERS |

**Supplementary Table S4: Predicted bridging sites without 3D modelling support.** Residues in bold are common to experimentally observed sites or to other predicted sites (Tables S1-S2). Highlighted residues in the last column are shared among Coronavirus proteins

| **Protein** | **Predicted site** | **Site alignment with MERS and Sars-Cov proteins** |
| --- | --- | --- |
| Nsp4—Nsp14 | C2989, C2997, **C6377**, H6380 |  |
| Nsp3—Nsp5 | **C1752, C1755,** C3301, **H3304** | 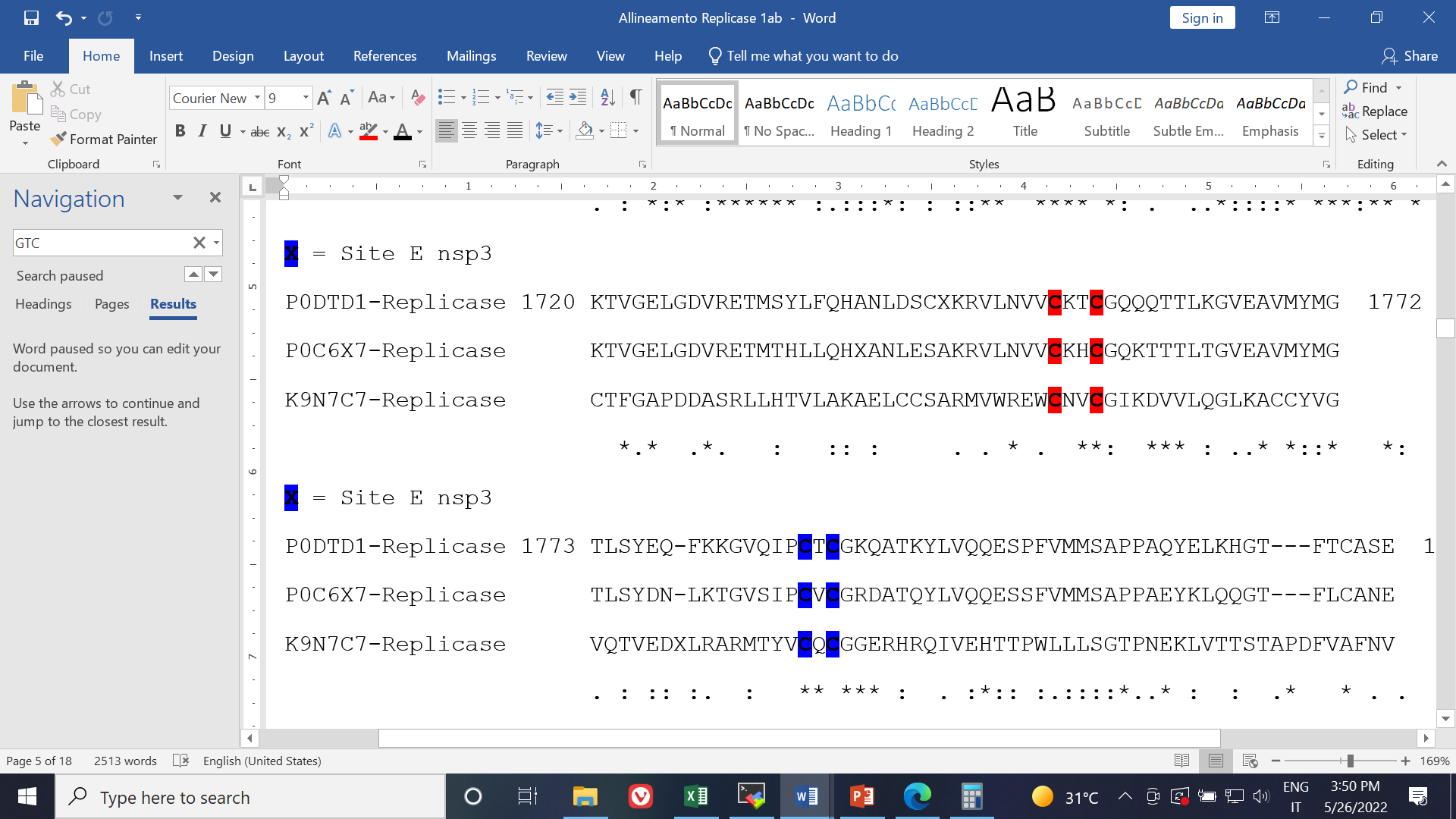  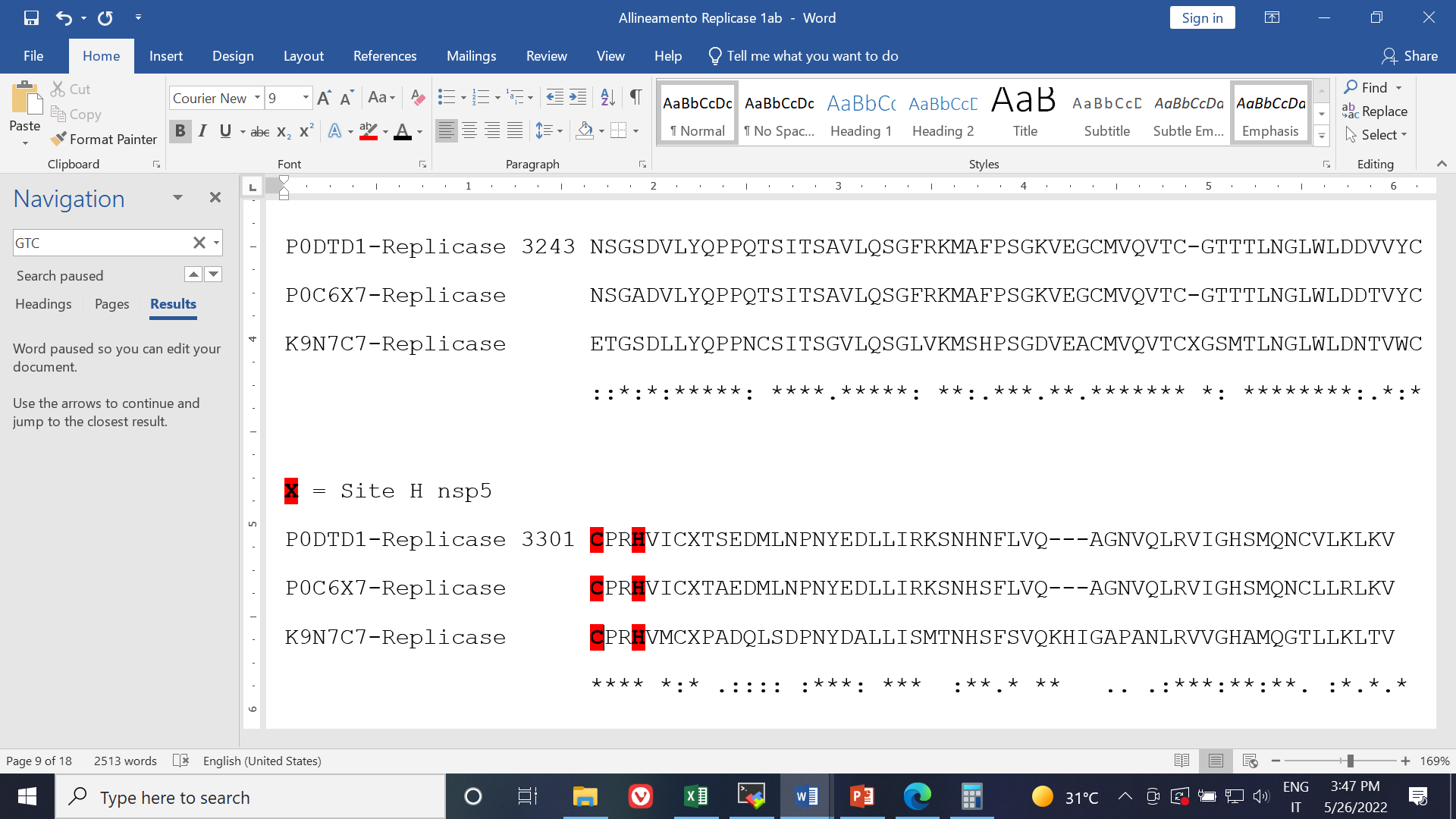 |
| Nsp2—Nsp13 | **C370, C373,** C5765, C5768 | 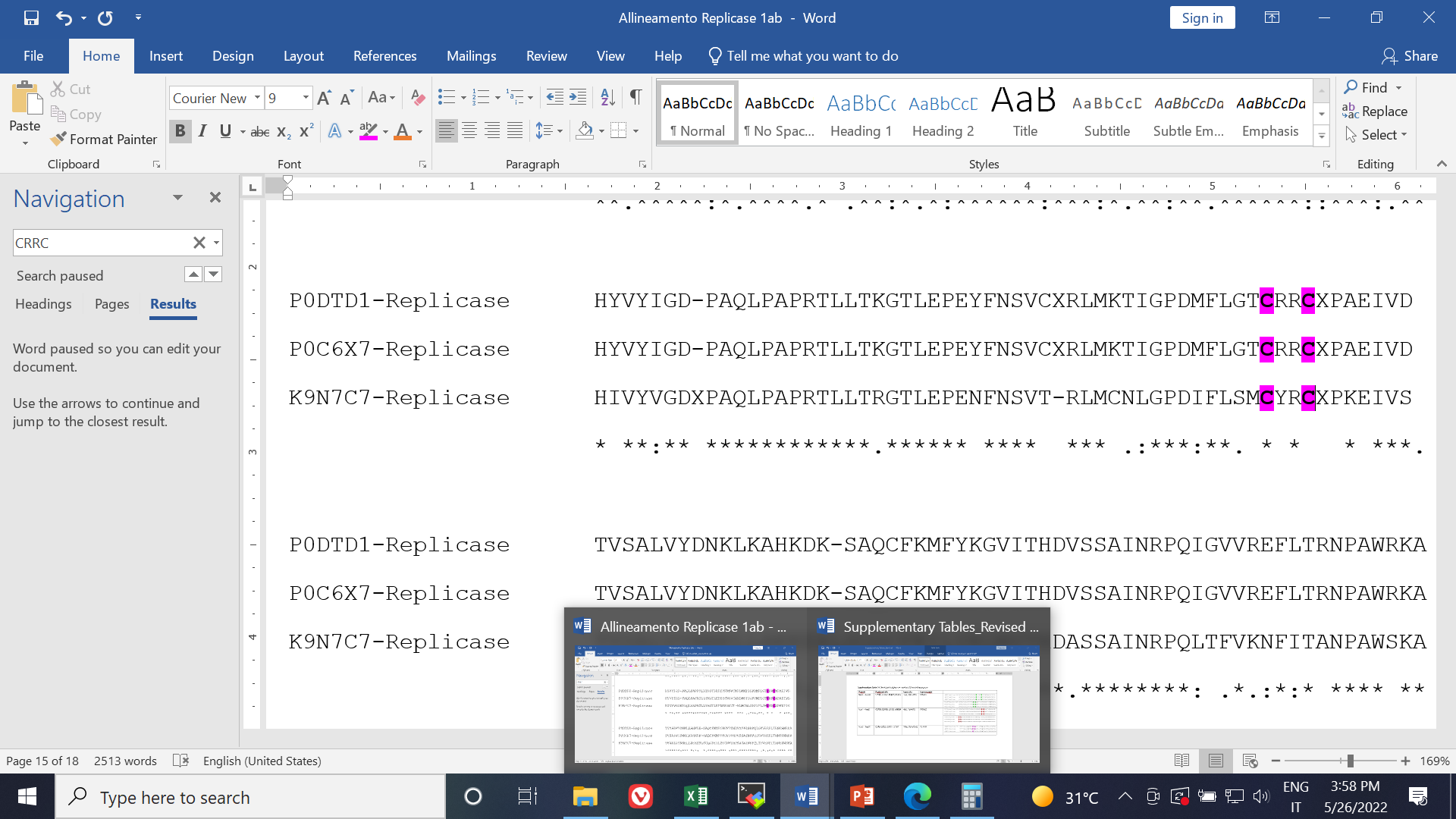 |
